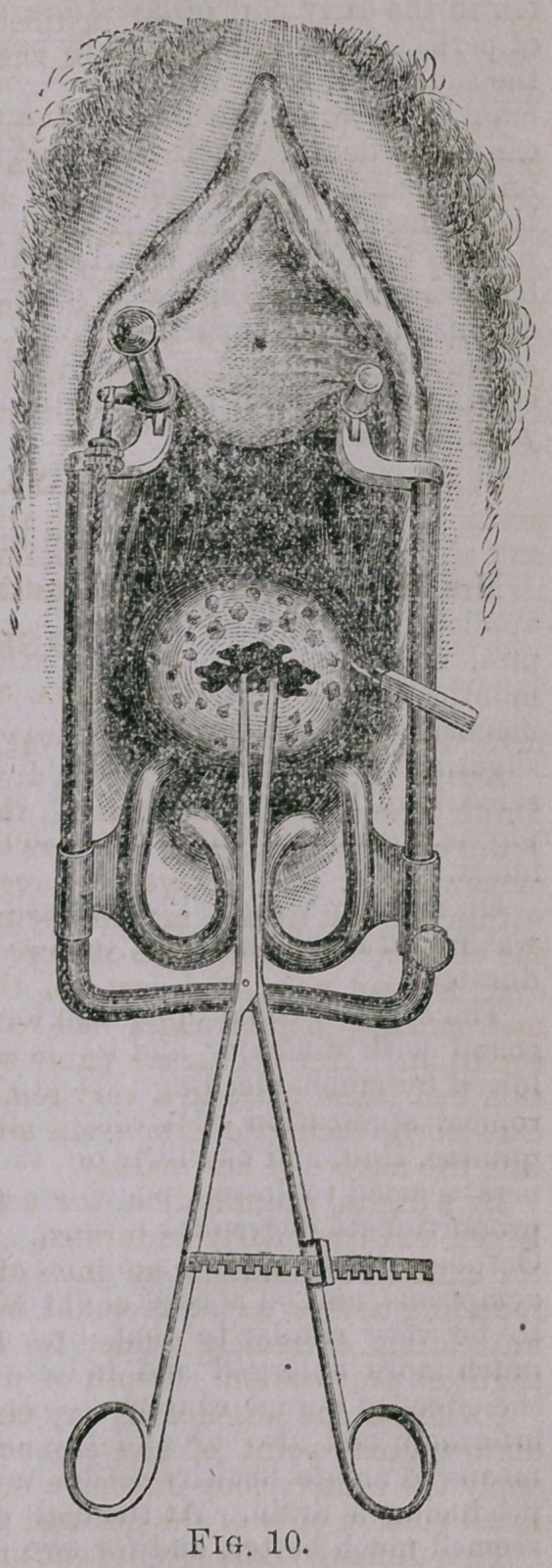# Clinical Notes on the Electric Cautery in Uterine Surgery

**Published:** 1873-01

**Authors:** J. Byrne

**Affiliations:** Surgeon-in-Chief to St. Mary’s Hospital for Diseases of Women; Clinical Professor of Uterine Surgery to Long Island Medical College, &c.


### Miscellaneous.

### Clinical Notes on the Electric Cautery in Uterine Surgery.

BY J. BYRNE, M. D.,

*Surgeon-in-Chief to St. Mary's Hospital for Diseases of Women; Clinical Professor of Uterine Surgery to Long Island Medical College, &c.*

In the preceding remarks it has been my aim to deal only with such questions as seemed to have a practical bearing on the subject of galvano-cautery; so that, for the sake of avoiding tedious details, many points of great interest and importance have been barely noticed, or passed over entirely.

With the same object in view, the clinical part of this paper will consist of a tabular record of operations, their subdivision into groups or classes, and such comments thereon as may serve to elucidate the more striking features of each, together with a few illustrative cases. The whole number of cautery operations thus far occurring within my own observation has been seventy-three, as follows:—

19 cases of epithelioma, including cauliflower cancer.
11 " encephaloid, or medullary cancer.
13 " catarrhal, inflammatory, and ulerative affections of the cervical canal of uterus.
5 " amputation of cervix (non-malignant).
4 " fibrous and fibro-cellular polypi.
4 " sessile fibroid tumors.
2 " deep ulceration of os and cervix.
1 " intra-uterine vegetation (non-malignant).

| | | |
|---|---|---|
| 2 | cases of | vascular tumors of urethra. |
| 4 | " | granular urethritis. |
| 3 | " | hemorrhoids. |
| 1 | " | perineo-vaginal fistula. |
| 1 | " | lipoma of scalp. |
| 1 | " | lipoma of cheek. |
| 1 | " | lipoma of ear. |
| — | | |
| 72 | | |

Of the thirty cases of malignant disease,

| | | | |
|---|---|---|---|
| 17 | were of the | uterus alone. | |
| 7 | " | " | uterus and vagina. |
| 3 | " | " | perinæum and vagina. |
| 1 | " | " | left labium. |
| 1 | " | " | clitoris. |
| 1 | " | " | breast. |

Among the nineteen cases of epithelioma,
7 were indurated or ulcerated only, and
12 were of the vegetating or cauliflower character. Of the latter,
7 were of the cervix uteri alone.
3 were of the perinæum and vagina.
1 was restricted to the left labium.
1 was restricted to the clitoris.

The following table shows the date of operation, the parts involved, and the condition of patients UP TO DATE, in seven cases of epithelioma in its ulcerating stage of development:

TABLE I.

| | Date of operation. | Parts removed. | Progress. |
|---|---|---|---|
| 1 | May 10, 1870. | Posterior lip. | Patient left hospital well, and though lost sight of since, believed to be cured. |
| 2 | July 7, 1871. | Entire cervix. | No return of disease; health entirely restored. |
| 3 | July 26, 1871. | Anterior lip. | No return of disease; died some months after from other causes. |
| 4 | Jan. 25, 1872. | Entire cervix. | No return of disease; general health entirely restored |
| 5 | Feb. 28, 1872. | Entire cervix. | No return of disease. |
| 6 | June 5, 1872. | Conical piece from centre of cervix. | Disease reappeared. |
| 7 | Nov. 13, 1872. | Entire cervix. | Operation at this time believed to be radically curative. |

There are two out of the above seven operations that demand especial notice—Nos. 6 and 7—the same patient being the subject of both, as also of a previous operation undertaken for the removal of a cauliflower outgrowth (see table II., No. 3). At the time this lady came under my notice, my experienee in galvano-cautery was comparatively limited, nor did I fully realize, though not without some misgivings on the subject, the great importance of removing tissues as far beyond those apparently implicated as can be safely done.

In every such instance, therefore, since met with, the removal of the outgrowth has been but the first part of each operation, except where, by traction being made on the tumor at a certain stage, a deeply cup-shaped stump could be insured. Indeed, from what I have since observed, I feel justified in believing that had this rule been observed in the above case, the result would have been entirely different.

As to the failure of a second operation in the case of this lady, my explanation is simply this: the anæsthetic used on the latter occasion was nitrous oxide gas, and owing to certain alarming symptoms manifesting themselves a few minutes after I had commenced to operate, I felt impelled, in my anxiety for the patient's safety, to stop much short of so complete and satisfactory an operation as I might otherwise have effected. However, as the patient's general health is yet good, the best results may reasonably be hoped for from the more thorough measures adopted within the last few weeks.

The following case (table I., No. 2,) bears so forcibly on the importance of effectually removing all the diseased structures at least, and at the same time so well illustrates my method of operating, that its introduction here may add to the interest of what has been said.

### CASE I.

### CARCINOMA OF UTERUS, involving both intra and supra-vaginal portions of the cervix.

About the 1st of July, 1871, I was requested by Dr. George K. Smith to see Mrs. ——, aged 47, the mother of three children, the youngest of whom being 10 years old. Previous to three years ago menstruation had always been regular; but since then, and up to within the last fifteen months, symptoms such as usually usher in the climacteric period were observed. The catamenia now, and for over a year past, had lost all the character of periodicity, and metrorrhagic hemorrhages had reduced her to a perfectly helpless condition. Her nocturnal pains were almost intolerable; emaciation had taken place to a very remarkable degree, and her anxious, care-worn, cachectic expression might alone have sufficed to indicate the nature of her malady.

By a digital examination, the cervix uteri was found much enlarged and irregularly indurated. The cervical canal was open to the extent of admitting an inch of the index finger, while the surrounding tissues, as far as could be reached within the neck, were unyielding, extremely tender to touch, and bled freely on the slightest provocation. Depth of uterus three inches.

Owing to the absence of any circular line of depression at the utero-vaginal point of convergence, it was found impossible to apply the cautery loop in such a manner as to include more than a small portion only of the diseased structures.

This difficulty, though not encountered before, had, nevertheless, been fully considered as one of the many contingencies likely to arise, and therefore, being anticipated, was provided for.

The patient having been anæsthetized, no trouble was found in bringing the diseased part into view, and by the aid of my speculum, ample space was afforded for any manipulation required.

The gentlemen present at this operation were Drs. G. K. Smith, Skene, Dwyer, and Bedell. The cervix was seized by a vulsellum held in the left hand, while with the cautery-knife* the cervix was slowly severed and removed without loss of blood. The same instrument, only more curved by being bent, was now applied to the deeper tissues of the cervix, which, while drawn down by a tenaculum, were cautiously sliced off piece by piece, laterally as well as upward, to the utmost extent deemed safe.

When the parts were thus quite scooped out, a deep bell-shaped cavity was left, from the bottom of which to the fundus uteri measured little over half an inch. No hemorrhage occurred during the whole operation.†

The recovery of this patient was no less rapid than remarkable, and if we except a very trivial secondary hemorrhage, and some degree of irritation arising from accidental scorching of the vaginal wall, no single inflammatory, febrile, or other complication turned up to mar her progress. She has become strong and robust, and up to a very late period has even menstruated regularly, the flow being of course but very slight, yet unaccompanied with pain or distress of any kind. She is under constant observation, and calls at stated intervals, according to my request, for the purpose of being carefully examined. There is but little of a uterus to be felt,

FIG. 10.

* The knife should be got into position before heating

† A microscopical examination showed cancer cells and free nuclei in abundance.

and the vaginal canal ends in a kind of cul-de-sac, at the bottom of which a still narrower passage may be detected, in depth about three quarters of an inch. The above report of this case was written in the early part of last June, and she is still in the enjoyment of perfect health, having safely passed the climacteric period. That the successful issue in the foregoing case is due to the thorough manner in which the diseased tissues were cored out, I think there can be no doubt. It is also suggestive of the probable causes of failures and disappointments so often met with by some of onr best gynæcologists, as referred to in the first part of this paper.

The following case clearly exemplifies the folly of trusting to half-way measures, and also where indurated and ulcerating conditions, however apparently limited in extent, resist judicious and active topical measures, how necessary it is to remove the whole cervix up to, and, if needed to insure success, to scoop out even beyond, the os internum.

### CASE II.

### CARCINOMA OF CERVIX.

Mrs. H——, aged 30 ; has had one child and two miscarriages ; applied for advice to the out-door department of St. Mary's Hospital in June, 1871. Menstruation had been regular up to six months before this date, but since then she has suffered from menorrhagia, sometimes excessive, but always prolonged, with shooting pains in the sacral and inguinal regions, and throbbing sensations in the vagina. She appeared much debilitated, and a physical examination of chest showed tubercular deposits in both lungs.

On making a digital examination per vaginam, the cervix uteri was found much tumefied, tender on pressure, and irregularly indurated.

The cervical canal in its inferior half, though open, admitted the sound with difficulty and its most careful introduction was followed by much bleeding. Depth of uterus three inches. By the topical application of strong solution of iodine and the use of quinine, iron, and cod-liver oil, the size of the cervix and its hardness seemed to lessen, while her general condition improved in a proportionate degree for a time, so that treatment was abandoned. October 4th she applied again on account of a return of her original symptoms, and on a careful examination her condition was found to be very similar to that first observed, and the uterine cervix much more enlarged and indurated. It was now decided to try the effect of actual cautery to cervical canal as far up as the os internum, and also around the os tincæ, hoping by such active means to create healthy action and perhaps relieve congestion by producing a drain. At the end of a month the local condition seemed much better, and for two menstrual periods following this last treatment she had no menorrhagia, and her general health appeared to improve.

This improvement, however, was but temporary, for she once more, on the 25th of January, reported herself as feeling much worse than ever, and an examination fully confirmed the truth of her suspicions. She stated that she had been flowing for two weeks continuously, as was very evident from her anæmic look, and on examination the diseased parts presented a much more tumefied and inflamed appearance than on any previous occasion.

It was now decided to remove the whole cervix by galvano-cautery. The condition of her lungs rendering the administration of an anæsthetic of doubtful propriety, and being also desirous to ascertain the amount and extent of pain attending such operations, she was induced to forego etherization. The operation may be described as follows: The uterus having been brought into view and steadied by means of my speculum, the cervix was seized with a vulsellum, and the cautery-knife, before being heated, applied posteriorly, the blade directed transversely, and its edge looking upward and forward. The battery being now immersed, the knife was carried completely around the circumference of the cervix close to its viginal insertion. In this manner a deep and somewhat oblique groove was made which served as a bed for the loop. The latter was now made to embrace the cervix still held in the grasp of the vulsellum, the battery again immersed, and some traction being made during the passage of the heated wire through the tissues, the operation was completed. When the cervix was removed, what remained of the uterus was deeply concave, and its cavity measured less than 1½ inch. There was no hemorrhage during or subsequent to the operation, and, what is of some consequence to know, she declared that the pain experienced during this operation was no greater than she suffered repeatedly before, when any active topical application was made.

May 16th. Menstruation has appeared twice since the operation, lasting each time four days, and without the slightest inconvenience or tendency to hemorrhage. She has gained flesh, is free from pain, and expresses herself entirely well. By a careful vaginal examination, no trace whatever of disease can be recognized either by sight or touch.

A microscopical examination of the part extirpated gave abundant evidence of carcinomatous disease.

As to the eleven cases of epithelioma characterized by exuberant outgrowths from a comparatively narrow base, the same tabular arrangement observed in the first class may be conveniently adopted.

The first case in this table possesses so many features of great interest, that any remarks beyond those embodied in the following history seem uncalled for.

### CASE III.

### EPITHELIOMA OF CERVIX UTERI.

Mrs. A ———, æt. 48, multipara, has always enjoyed the best of health up to within a few weeks of my being called to see her, which was in July, 1869. She complained of great back-ache and bearing down sensations, and noticed some discharge of mucus occasionally mixed with blood. Menstruation regular and normal in character and has always been so. By digital examination the cervix was found much tumefied, more particularly the posterior lip, and painful to touch. On inspection by speculum, there was found a slightly elevated and velvety-looking surface stripped of epithelium and extending over at least one-half of the posterior lip. Anterior half of cervix, though somewhat swollen, yet soft to the touch and paler. The local treatment consisted in warm vaginal douching and the application of iodo-glycerine to the diseased parts once and sometimes twice a week. A marked improvement was noticeable after a few weeks of this treatment, and hopes were entertained that it might be permanent. November 9th, I was requested to see her again, when she stated that her old pains and other disagreeble symptoms had lately returned, but in a much more severe degree. Besides, there was this peculiarity, she said, about her sufferings, that she was seized about four or five o'clock every morning with severe lumbar and hypogastric pain, which lasted up to nine or ten, but after this latter hour she felt relieved and continued comfortable until the same hour of the succeeding morning A speculum examination now revealed a similar condition of the uterus to that first observed, when the same active topical measures were once more resorted to, but on this occasion with no improvement whatever.

TABLE II.

VEGETATING EPITHELIOMA.

| | Date of operation. | Parts removed. | Progress to date. |
|---|---|---|---|
| 1 | Jan. 4, 1870. | Posterior lip. | No return of disease. |
| 2 | Dec. 4, 1870. | Left labium vagina. | No return; died from other other causes 16 months after operation. |
| 3 | June 18, 1871. | A part of both lips which the tumor seemed to involve equally. | Reappeared in ulcerating form. |
| 4 | Sept. 13, 1871. | | Died from causes not attributable to operation. |
| 5 | Nov., 1871. | Labium and part of perinæum. | Little gained by operation. |
| 6 | Feb. 11, 1872. | Tumor and cervix. | Had recovered perfectly and no return of disease at last report. |
| 7 | April 26, 1872. | Entire cervix, | No return of disease. |
| 8 | March 6, 1872. | Clitoris. | Believed to be cured. |
| 9 | May 4, 1872. | Entire cervix. | Well at last report |
| 10 | July 20, 1872. | Entire cervix and cauterized, suspicious, warty looking excrescences on vagina. | Doing well at last report. |
| 11 | Nov. 20, 1872. | Left labium and nates. | Greatly relieved. |
| 12 | Dec. 4, 1872. | Left labium and nates. | Greatly relieved. |

The disease, for some time suspected, was diagnosed as epithelioma, and for the following among other reasons: 1st. The hereditary predisposition existed in a marked degree, and of which important fact I had had some personal knowledge ; and 2d. The disease had resisted treatment, constitutional as well as local, well calculated to improve if not to cure a less serious ailment.*

About this time (December, 1869), Dr. Noeggerath exhibited at a meeting of the New York Obstetrical Society a specimen of epithelioma removed from the cervix uteri by galvano-cautery, and its resemblance of what I had observed in my own patient induced me to request a consultation with Dr. N.

This having been readily assented to, and my diagnosis concurred in, it was decided to remove the diseased part by galvano-cautery, and the operation was performed on the 4th of January, 1870.

This was accomplished by the platina loop, the shape of the tumor rendering this a work of no great difficulty, and the part excised embraced nearly the entire vaginal portion of the posterior lip.

A little glycerine and tannin was brushed over the cauterized surface, the bowels kept quiet by opiate suppositories for a few days, and in less than two weeks the patient, with no other local treatment save tepid vaginal baths, was up and about. On the 1st of February, 27 days after the operation, the healing process was found to be so nearly complete that nothing further seemed needed, and being entirely relieved of all pain she rapidly recovered strength.

A careful speculum examination, however, was made every few weeks for some time, with a view of detecting any reappearance of the disease, but nothing of the sort being noticed, these precautionary measures were abandoned after a few months. About a year ago this lady requested me to see her again, and stated that she had suffered so much from pelvic pains, referable particularly to the bladder and pelvic region, that she feared there might be some return of her former difficulty.

A careful examination failed to detect anything more than a slight abrasion on the posterior aspect of the os tincæ, and a certain degree of firmness of the part from which the disease had been removed. This, however was attributed to mere congestion of the part, and active topical applications quickly restored the parts to their natural condition.

Other measures calculated to relieve slight cystitis, which undoubtedly caused much of the pain and distress complained of, were prescribed, and the patient rapidly recovered. Six months ago (May 20, 1872), I was requested to see her, when she informed me that she began to feel a little anxious on account of some slight mucous discharge and more or less tenesmus. It was deemed best to examine into the state of the uterus; this was found to be in a condition precisely similar to that of twelve

* This latter fact is, in my opinion, of the utmost importance in all such cases, and presents strong presumptive evidence of malignancy.

months previously, and after two applications of iodo-glycerine to cervical canal, the improvement at last examination was so great that no further treatment was called for.

Menstruation has not appeared regularly, and this last attack was doubtless due to a scanty flow at a previous period. It should have been mentioned that the outgrowth when removed was submitted to a careful microscopical examination, and all the evidences of true epithelioma were present. It is now nearly three years since the operation.*

### CASE IV.

The second case in the preceding class was one of extensive epithelioma, involving the whole of the left labium vaginæ in an old lady aged 70. The entire part was removed by cautery, and in less than one month from the date of operation the surface healed and seemed to be covered over by perfectly smooth and healthy material. She continued to enjoy good health, for one of her years, during the succeeding twelve months, when symptoms indicating cancerous disease of mesentery and other internal parts rapidly became developed, and from which she succumbed sixteen months after the operation. It is worthy of remark, however, that the surface from which the diseased mass had been excised remained perfectly healthy up to the time of her death, nor were any of the pelvic organs concerned in the final work of destruction.

### CASE V.

The case having already been referred to (No. 5, Table I.), calls for but little further notice. This lady, whose age is thirty-one, a widow, consulted Dr. J. Marion Sims, in consequence of having been informed by her physician that she had cancer of the womb, which she did not believe, at the same time giving as a reason for her opinion the fact of her having had little or no pain or uncomfortable feeling in that region; and, moreover, that after full inquiry she felt satisfied there was no hereditary predisposition to such a disease. Menstruation had always been regular, and she had had no hemorrhage, but during the menstrual intervals she had of late noticed some watery discharge of an offensive odor.

Dr. Sims recognized a large cauliflower mass springing from the whole circumerence of the cerfix, and spreading out so as to occupy a great part of the vaginal cavity. He advised its removal, and requested me to operate by galvano-cautery, which I did on the 18th of June, 1871. In this operation the neck of the tumor was embraced by the wire loop and its removal thus effected; but in addition to the mistake of leaving too much behind, as before stated, there was another error committed, which, on account of the clinical lesson it teaches, ought not to be overlooked. The

* A few weeks ago (Nov. 26) the uterus was carefully examined, but no evidence of a return of the disease could be detected.

instrument shown in Fig. 2 was then new, and used on that occasion for the first time, so that I was not accustomed to this improved means of contracting the loop, and miscalculated as to the screw motion. The consequence was that the tissues were too rapidly severed, and though there was no loss of blood whatever at the time, an alarming secondary hemorrhage took place about thirty-six hours after the operation, requiring the use of tampon.

No. 4 is a case where I assisted Dr. James L. Brown in operating, and which has been reported elsewhere. This was a promising case, and its fatal termination had nothing whatever to do with the merits of the operation, death being caused mainly by imprudence on the part of the patient, and other circumstances beyond the control of her medical adviser.

The patient, in whose case parts of the right labium and perinæum were removed on three occasions (Nos. 5, 11 and 12), is the wife of a physician in this city. The cautery was resorted to in this instance merely for the purpose of excising portions of a large suppurating and offensive mass, hoping thereby to contribute in some measure to her comfort, or rather to modify her suffering.*

The extent to which the rectum, vagina, and neighboring parts were involved, was such as to render the case an utterly hopeless one, and consequently nothing beyond palliative effects could be looked for from any operative proceedings.

### CASE VI.

On the 11th of last February I was requested by Dr. J. Marion Sims to operate by galvano-cautery in the case of a lady whose history is as follows: Mrs. ———, aged fifty, of healthy ancestry on her father's side, but several members of her mother's family have died from pulmonary affections, and one, an aunt, from cancer of breast. Menstruation commenced at 14 and has always been regular up to February, 1871. Has had seven children, and a premature confinement in 1856, from which she recovered speedily. From February, 1871, until August, the catamenia were absent, but in the latter month she had a profuse metrorrhagia lasting for several days, and returning more copiously three weeks later.

On examination per vaginum, a tumor about the size of a hen's egg was found springing from the cervix and projecting into the vagina; canal of uterus of normal depth; body not hypertrophied. This tumor was removed by ecraseur on September 23, 1871, and presented under the microscope the characteristic appearance of epithelial cancer. The patient seemed to improve in some respects until about the first of January, 1872, when hemorrhage returned and large quantities of blood were lost throughout that whole month.

Dr. Sims saw her on the 10th of February, and discovered a

* Dr. Geo. M. Beard has also operated previously in this case by electrolysis, with but little effect.

large cauliflower tumor springing from the cervix and completely filling up the upper half of the vagina. The following day, February 11th, was appointed for its removal, and Dr. S. having accidentally sprained his ankle while stepping out of his carriage, requested me to see her and operate for him. The patient was found to be in a very exhausted condition from loss of blood, and emaciated to so remarkable a degree that grave doubts were entertained as to the propriety of operating or risking the administration of any anæsthetic.

In such a state of things, however, some interference seemed urgently demanded, and ether having been administered, the operation was proceeded with in the following manner:

The platina loop was with considerable difficulty made to embrace *the upper circumference of the cervix*, and when *moderately tightened* the battery was immersed; little or no contraction of the loop being effected for a few seconds, so that the superficial tissues of the part to be cut might be thoroughly cauterized. When the wire was supposed to have entered the tissues a quarter of an inch or thereabouts, firm and steady traction was made on the tumor by means of a vulsellum,* and its connections *very slowly* severed by a further tightening of the loop. By this manœuvre the surface from which the tumor had been removed presented a deeply concave appearance and there was no hemorrhage whatever. The uterine cavity measured about one inch from the bottom of the wound. No topical application was made.

As this patient resided some distance from the city, I had no opportunity of observing her subsequent progress; but one of the gentlemen who assisted at the operation † informed me some days after, when he called to see her, that her condition was very precarious. Towards the end of May, having occasion to visit her neighborhood, I called to see her, and found her going about and able to superintend her household affairs.

The following reply to a note of inquiry has been since received from her attending physician, Dr. Furgang, of East New York:

"Dear Doctor: In accordance with your request I have given Mrs. ——— a very careful examination. Her pelvic organs, or what is left of them, seem to be in a perfectly healthy condition. There is nothing to the touch or sight that would lead to the suspicion of a return of her disease. The part from which the tumor was taken is a little puckered, but soft and covered with healthy-looking mucous membrane, and there is no tenderness on pressure there or in any of the adjoining parts. Her appetite is excellent, she sleeps well, and is rapidly gaining in strength and flesh."

This case calls for no further comment.

---

* Traction by the cautery instrument should, in *all* such cases, be carefully avoided and the instrument kept steady and in the same position from the beginning to the end of the operation.

‡ Dr. Nichol.

### CASE VII.

This was what appeared to me to be epithelial cancer of the clitoris, though my friend Dr. J. C. Nott, who was present at the operation, thought it might possibly be non-malignant, and such as J. Y. Simpson has described under the term of "caruncle." The tumor was about the size of an English walnut, had all the characteristic appearances of vegetating epithelioma, and required but a few months for its development. It was removed by means of the cautery-knife (Fig. 3), and the patient left the hospital well, but has not since been heard from.*

### CASE VIII.

### VEGETATING EPITHELIOMA INVOLVING THE WHOLE CERVIX.

For a full report of this interesting case, of which the following is a synopsis, I am indebted to Dr. C. H. Giberson:

Mrs. ———, aged 32, the mother of two children, and a widow for ten years; eldest child healthy, but the younger, now ten years old, has spinal curvature. She says a married sister died at 36, of "what was called cancer of the womb." Has had almost constant hemorrhage for the past thirteen months and seems to grow steadily worse, until now (April 15th, 1872), she is very anæmic and much depressed in spirits.

April 23d she was examined by me and the condition found to coincide with the above description. April 26th, the tumors and cervix were removed by cautery, much in the same manner as that detailed in case No. 6, but with this addition, that after all that could be embraced within the loop had been taken away, suspicious spots on the vaginal duplicature were excised by means of the cautery-knife. When the operation was completed the uterine cavity measured 1½ inch.

May 10th. Wound presents a healthy granulating appearance.

June 1st, five weeks after operation, healing process going on rapidly; uterus measures two inches in depth, the increase being due to filling up of deep cavity made by cautery.

June 20th. Dr. Byrne examined her and found a small granulating surface and looking well. Iodo-glycerine applied to surface. First menstruation since operation appeared June 8th and lasted moderately three days.

July 31st. Uterus 2¼ inches deep, os small, no leucorrhœa, vaginal and uterine surfaces smooth and soft, very slight point to right of os of granular appearance. General health good, but complains of shooting pains in lower abdomen.†

September 30th. Third menstruation, lasting three days, has passed over without trouble.

---

* Two operations were resorted to in this case, within the last month, tearing away, each time, large masses of suppurating vegetations and thoroughly cauterizing the subjacent surface.

† The increased depth of the uterus, as noticed at this examination, is due to a filling up of the excavation by *healthy* granulation, and is not peculiar to this case,

October 12th. Considerable pain and slight occasional flow during the past ten days until yesterday, but vaginal examination shows no ulceration and no induration perceptible.

Since the above report (October 12th) the patient is doing well, but it is evident that her case is a less promising one than could be hoped for, and hence I have thought proper to present it as a darker side of the picture.

She has no cachectic appearance, however, but on the contrary looked to me so much stronger and healthier, when seeing her in the street two or three weeks ago, that I hardly recognized her. Nevertheless I look forward to her future history with much interest and some little misgivings.

### CASE IX.

### VEGETATING EPITHELIOMA INVOLVING THE WHOLE CERVIX.

Mrs. ———, aged 45, has had seven children and two miscarriages; the last living child seven years old. Menstruation has always been regular up to six months ago, when the flow became excessive and the interval less and less, until now (April 18th, 1872,) it is almost continual. On digital examination, the whole of the cervix uteri was found very much enlarged and greatly indurated, but soft and spongy on its presenting surface, tender to pressure, and bleeding on the slightest touch. The body of the organ was not enlarged and the vaginal walls intact.

When brought into view the os was observed to be surrounded by what appeared like luxuriant granulations, though the unstripped parts of the cervix were in color somewhat paler than normal. The case was diagnosed as one of epithelioma in the early sprouting stage, and she was admitted into St. Mary's Hospital for operation May 4th. The patient was anæsthetized and the entire cervix removed by the cautery, but the method pursued being so entirely similar to that of other cases already detailed, no further description is here called for. There was no blood lost during the operation, nor was there any secondary hemorrhage. Vaginal bathing with tepid water and carbolic acid was commenced on the third day after operation and continued for two weeks; sixteen days after the operation a speculum examination was made, and the surface from which the disease had been excised was almost entirely covered with healthy membrane, and the patient, feeling well and anxious to see her family, was permitted to leave the institution. She has not since been heard from.

Case No. X., being very similar to the above, offers no points of special interest to warrant a full report on the present occasion, and sufficient time has not yet elapsed to say anything of results, further than that they are not less promising than in any of the preceding cases.

Case No. XI. is that of the patient whose condition has been noticed (No. 4), and this second operation, like the former, was resorted to merely for the purpose of taking away such parts of the suppurating excrescences as could be safely spared.

With regard to the eleven cases of carcinoma in which, like the above, operative measures were resorted to for the purpose of affording temporary relief merely, the limits of this paper will not permit of their being referred to at any length. In seven of this latter class the disease had attacked both vagina and uterus to such a degree as to almost obliterate the one and utterly degenerate the other; yet in no single instance did the removal and destructien of such diseased tissues as could be safely reached fail relieve in a very remarkable degree, and add to the comfort of these afflicted sufferers.

This single statement, it seems to me, supported as it is by actual observation, ought to satisfy those who question the utility of any operation in such hopeless conditions. It is certainly no principle of conservative surgery to ignore palliative measures, even where disease is admittedly incurable; and yet, among the numerous victims of this terrible destroyer, how many a valuable life that might have been safely prolonged and robbed of much of its wretchedness has been allowed to ebb away in loathsome torment!

It is true, until very recently, non-interference in uterine cancers has been justifiable and eminently proper, owing to a want of the means whereby such ailments could be safely ameliorated, but I am fully convinced by past experience that this want no longer exists. However transitory, therefore, the relief may often be, I doubt the wisdom of those who in the face of facts would still persist in thinking that their whole duty had been performed by quoting a hackneyed axiom in the pathology of these diseases, which says: "When the patient's constitution has really become infected, these diseases, if extirpated, invariably return and conduct the person who is affected by them to inevitable destruction."*

It should not be forgotten, however, that in very many instances the prolongation of life but for one month may be of the highest consequence to a family about to be deprived of a mother's influence and watchful care, even though that mother be a helpless invalid.

Furthermore, in order to determine as to the propriety of operations for the relief of such patients, there are, or ought to be, but two questions worthy of consideration, namely: Have we the means whereby such a course may be undertaken without risk to life, or in any way adding to existing evils? And secondly: Have we good grounds, *i. e.*, clinical data, for hoping to ameliorate the sufferer's condition thereby? Appropos of these considerations I submit the following case:

### CASE XII.

### CARCINOMA OF UTERUS AND VAGINA. OPERATION PALLIATIVE.

Mrs. ———, widow, aged 30, has two children, and always en-

* Muller on Cancer, etc. London. 1840, page 28.

joyed perfect health until some time in the month of January last. About this time menstruation, previously regular, appeared in great excess and lasted over eight days. This was followed by a copious watery discharge for two weeks, when metrorrhagia again appeared and hemorrhage on the latter occasion continued for ten days. A watery and whitish discharge as in the previous interval continued up to the first week in March, when, after a hard day's work as chambermaid in a hotel, she was seized with violent expulsive pains and almost fatal hemorrhage. She cannot remember how long the flooding lasted then, but on its ceasing she applied for admission and was received into one of the New York hospitals, where she remained for a few weeks without having had anything done for her. On Friday, the 10th of May, she applied at the College of Physicians and Surgeons in 23d street, and was examined by Professor Thomas, who at once discovered extensive carcinoma of the uterus, involving the vaginal walls anteriorly and posteriorly, and accordingly pronounced her case as utterly hopeless, which it certainly was. Under these circumstances she applied for admission to St. Mary's Hospital, May 13th, 1872, with a letter from Dr. Charles S. Ward, who stated that he recommended the patient to see me, in hopes that I might be able to do something towards relieving her temporarily by galvano-cautery.

When admitted, she said she had not ceased flowing for several days past, and her wretched and bloodless countenance bore fearful testimony to the truth of this statement, for she was unable to move one step without support, and it was found necessary to administer stimulants freely before she could be safely removed to bed.

By digital examination I found the condition precisely as Dr. Ward had stated, and as the loss of blood was frightful, nothing could then be done beyond tamponing the vagina. This succeeded in arresting the hemorrhage; but on its being removed the following day it was evident that something of the kind would again be necessary, and a fresh tampon was applied. This latter was allowed to remain in 48 hours, and its removal not being followed by any return of hemorrhage, I decided to try what could be done by the cautery at the earliest possible moment.

The operation which took place on Saturday, May 18th, may be described as follows: The upper half of the vagina being packed with a large encephaloid-looking mass adherent on all sides, it was found impossible to loop more than a portion of it, so that after removing all that could be taken in this way a much larger proportion yet remained. The soft brain-like character of the outgrowth preventing the heated wire from acting as a hæmostatic, considerable blood was lost, and it was therefore determined to complete the operation as quickly as possible. This was done by grasping the more projecting parts of the mass by a strong polypus forceps and forcibly tearing them away piece by piece, until the greater part of the spongy excrescence was twisted off from the uterine cavity as well as the vagina. The cautery-knife was employed to trim off and scoop out whatever remained, and the dome-shaped cauterizer thoroughly applied to the whole subjacent surface. It was now found that the hemorrhage had entirely ceased, but as a security the uterine cavity and vagina were carefully tamponed and the patient put to bed.

Her daily record for the succeeding two weeks contains nothing of sufficient importance to warrant minute details. The tampon was removed 48 hours after the operation, and no hemorrhage whatever appearing, the vagina was ordered to be washed out twice daily with a mixture of carbolic acid, glycerine and water.

No peritoneal or other inflammatory trouble followed this operation, and very many of her former pains and distressing symptoms were entirely relieved. Her appetite and sleep returned, and in three weeks she was strong enough to sit up and walk through the ward.

The purulent discharge following the use of the cautery continued for 15 days, after which appeared a slight, serous-looking, but yet entirely inodorous drain.

June 15th, the parts operated upon were carefully examined and found to be smooth, but uneven and somewhat hard to the touch, but as far as the eye could reach, seemed to be covered with some kind of membrane, and manipulation provoked no hemorrhage. A steady improvement has been observed in her appearance from day to day, and now feeling comporatively strong and being anxious to visit her friends, she was permitted to leave the hospital. I regret to add that I have not been able to trace her whereabouts since.

Cases, of which the preceding one may be considered a type, might also be related, had I not already far exceeded the proposed limits of my remarks. I deem it proper to state, however, that in three out of the ten cases of pelvic encephaloid cancer operated upon, the disease, though limited, included the whole uterus, and these were by far the most unsatisfactory of this class. In one case, a patient of Dr. Sims, I operated twice, and though in the second effort, he, Dr. S., scooped out large quantities of the diseased mass from the uterine cavity by means of his curette, preparatory to the application of the cautery, and despite a very complete charring of all the denuded surfaces within reach, the bleeding excrescences were rapidly reproduced. This lady, who resides in another State, though not improved by what had been done, was certainly made no worse, and in accordance with advice returned to her home.

Altogether, from what I have observed in these three cases, I believe but little if any advantage can arise from the use of the electric cautery in carcinoma of the body of the uterus, when this organ has been the starting point of the malady, and when the cervix has already been destroyed by the disease in its upward march.

The next case to which I shall refer is one of interstitial fibroid or perhaps what might more properly be designated diffuse fibrous hyperplasia of the right half of the uterus.—*Medical Record.*

## Figures and Tables

**Fig. 10. f1:**